# COVID-19 pandemic and the management of type-2 diabetes mellitus among women in Fiji: concerns and context specific interventions

**DOI:** 10.3389/fcdhc.2026.1846673

**Published:** 2026-09-11

**Authors:** Eunice Okyere, Kissinger Marfoh, Ramneek Goundar, Litia Makutu, Ditoga Kabukeinamala

**Affiliations:** 1 Department of Public Health, School of Public Health and Primary Care, College of Medicine, Nursing and Health Sciences, Fiji National University, Suva, Fiji; 2 Department of Epidemiology, School of Public Health and Primary Care, College of Medicine, Nursing and Health Sciences, Fiji National University, Suva, Fiji

**Keywords:** coping strategies, COVID-19 pandemic, diabetes management, Fiji, interventions, qualitative research, women

## Abstract

**Introduction:**

The COVID-19 preventive strategies had a profound impact on type-2 diabetes management, creating challenges for individuals who depended on routine in-person consultations with healthcare workers. Given that the pandemic did not affect diabetic women equally across countries worldwide, this research was conducted in Fiji to explore how women living with type-2 diabetes managed their condition during the COVID-19 pandemic.

**Materials and methods:**

This study used a qualitative approach from a descriptive phenomenological lens. A total of 45 women with type-2 diabetes were purposively selected for in-depth interviews, as the study depended on participants ability to provide relevant information on the phenomenon being investigated. The data collection involved using a semi-structured interview guide with participants selected from different ethnic groups, ages, religions and settings to enhance data diversity. The data were analyzed using an inductive thematic analysis approach.

**Results:**

The themes that emerged were the perceived impact of the COVID-19 pandemic on T2DM management, coping strategies and recommended interventions. The perceived impact of the COVID-19 pandemic on diabetes management included limited access to healthcare workers and diabetes supplies, canceled or postponed appointments, changes in physical activities, difficulty in maintaining standard diets and diabetes complications. The coping strategies identified were emotional eating, religious practices, family support and the use of herbal medications. Participants recommended the need for financial support, accessible specialized healthcare services, intensifying diabetes education, virtual clinics and providing door-to-door diabetes supplies in crises, like the COVID-19 pandemic.

**Conclusion:**

The findings underscore the need for stakeholders to mitigate the pandemic’s impact on women with type-2 diabetes by ensuring better access to healthcare services, providers, and essential supplies to improve disease management. To ensure continuity of care during emergency situations, policy makers and health managers should implement interventions including virtual clinics and home deliveries of diabetes supplies. Strengthening education on diabetes management can prevent complications by addressing behavioral and safety issues such as emotional eating and the unauthorized use of herbal medications.

## 1 Introduction

COVID-19 disproportionately affected people with chronic diseases with an increased risk of hospitalization and mortality (1). Diabetes mellitus has emerged as a distinct comorbidity that is linked with severe disease and increased mortality in COVID-19 (2, 3). Patients with diabetes are more susceptible to SARS-CoV-2 infection, which is subsequently linked to an increased risk of new-onset disease (4, 5). However, conflicting evidence suggests that mild and asymptomatic infection are not associated with glycemic progression (6). The SARS-CoV-2 virus induces hyperglycemia, subsequently leading to immune dysfunction, increased vulnerability to infections and the development of long-term diabetes complications (7, 8). Individuals with diabetes are more likely to develop long COVID and experience long-term consequences than those without the condition (9). In the initial phase of the COVID-19 pandemic, people with type 2 diabetes (T2DM) accounted for approximately one-third of the COVID-19 deaths (10). The Fiji government aligned with global health guidance, classified individuals with diabetes as high-risk group. This classification, combined with restrictions such as lockdown, social distancing and isolation could increase stress, anxiety and negatively impact T2DM self-management (11). Studies have identified high prevalence of anxiety and depression among individual with T2DM compared to the general population, linking these conditions to reduced diabetes self-management, increased risks of complications and premature death (12, 13).

The pandemic interfered with regular T2DM services because many healthcare workers were reassigned to manage COVID-19 related issues, leading to a considerable reduction in in-person appointments, in various countries, including Fiji (14, 15). Many nations, particularly developed ones, replaced in-person visits with virtual care to provide safer, convenient, and timely medical access with lower risk of transmission (16–18). Telemedicine consultations increased during the pandemic, and most participants were satisfied with the experience (19).

Scholarly research regarding lifestyle changes during the COVID-19 pandemic has revealed contradictory findings in dietary and physical activity behaviors among individuals with T2DM. While some adopted healthy eating patterns (15, 20), others experienced increased consumption of unhealthy foods and reduced physical activities (21, 22). The increased in food consumption was attributed to increased time at home, boredom, movement restrictions, and reduced physical activity due to infection concerns (22). These studies were conducted in developed countries such as Italy, Poland, Denmark, Japan and Spain (20–24). Given the limited information on developing countries like Fiji, this study addresses significant research gap.

Diabetes is the leading cause of death in many developing countries, with incidence expected to rise due to the impacts of the COVID-19 pandemic (25, 26). It also remains the primary cause of premature death in the Pacific Islands Countries, including Fiji (27). These countries, represent nine out of ten nations worldwide with the highest prevalence of diabetes and obesity in women (28). An estimated thirty percent of the Fijian population is diagnosed with diabetes, with T2DM being the most prevalent. Consequently, diabetic foot amputations have increased to approximately one every eight hours (29, 30). The geographical position of Fiji poses challenges to ensuring equitable health service delivery and accessibility, for its diverse population. The situation is further exacerbated by a vast maritime landscape, climate change and natural disasters (31, 32).

The Fiji government describes diabetes as a crisis, as its burden extends to individuals, families, and social and work life. Managing T2DM properly during emergencies like the pandemic, can minimize the burden of comorbidities, enhance glycemic control, and reduce the risk of diabetes complications (12, 33, 34). Failure to do so may result in severe complications including disabilities that negatively affects patients quality of life (35, 36). Recent studies highlight increased sex-based disparities in T2DM, showing higher rates of depression, anxiety and cognitive limitations in women, alongside higher prevalence of coronary heart disease, stroke and mortality (37, 38). A nationwide population-based cohort study of Norwegian patients with T2DM during the pandemic revealed that, women were more psychologically affected by the pandemic, with physical inactivity, weight gain and severe mental health effects (39). Though women with T2DM were among the high-risk groups during the COVID-19 pandemic, no study has been conducted to explore the impact of the pandemic on the management of T2DM in Fiji, hence the essence of this study. Therefore, this current study seeks to answer the following questions:

1. What are the perceptions of women regarding the management of T2DM during the COVID-19 pandemic?
2. What coping strategies did women use to manage T2DM during the COVID-19 pandemic?
3. What interventions can improve T2DM management among women during the COVID-19 pandemic?

This study provides valuable knowledge to support women with T2DM in emergency situations such as the COVID-19 pandemic. It contributes to health literature, by offering insights for healthcare managers, health workers and other relevant stakeholders to address the short- and long-term negative impacts of the pandemic on women with T2DM. Furthermore, the findings highlight interventions that, when implemented could improve T2DM management within the study area.

## 2 Materials and methods

### 2.1 Study design

This study used a qualitative method with a descriptive phenomenology design. The qualitative approach was chosen due to its strength in obtaining deeper, richer and more detailed data from the participants in real life situations (40). Phenomenology explains shared meaning of people’s experiences in relation to a phenomenon with the aim of revealing the essence of perceptions regarding these experiences (41). A descriptive phenomenology study design allows researchers to identify factors that relate to the phenomenon and to assess their impact on individuals involved (42). As such, a qualitative descriptive phenomenological approach enabled the researcher to delve into the lived experiences of women with T2DM during the COVID-19 pandemic in Fiji.

### 2.2 Recruitment and setting

This study was conducted in Suva and its neighboring suburbs including Lami, Nasinu, Nausori and Lami. Choosing participants from across these different settings, enhanced the study area diversity and allowed the researchers to gain a deeper insight into the phenomenon investigated (43). Suva is the capital of Fiji and the largest metropolitan city. It is situated on the southeastern coast of the island of Viti Levu, located in Rewa Province in the Central Division. Participants were recruited from these communities as part of a larger study investigating the impact of the COVID-19 on women.

The study participants consisted of 45 women with T2DM. The investigators considered factors such as age, marital status, ethnicity and occupation to select participants for the study, thereby improving diversity in the data collected. Participants were contacted directly through email or phone calls to request their participation. Information sheets containing comprehensive details of the study, including the study’s purpose, aim, benefits and procedures, were given to the research participants. This enabled them to make an informed voluntary decision, about participating in this study (44). Interested participants contacted the researchers who visited to determine their eligibility using the inclusion criteria including women with T2DM within the study area, 18 years and above, without mental disorder and willing to participate in the study.

Purposive sampling was used to intentionally select participants with specific knowledge and experience essential to the study. This non-probability technique allowed the investigators to select participants to provide rich, in-depth information, to answer the research questions. Purposive sampling relies on researchers’ discretion to select participants from the study population (45). However, it is recommended for researchers to be informative about the purpose of the study taking into consideration, the inclusion criteria (46). This enabled the researchers to explain the impact of the findings on the population.

### 2.3 Data collection technique and instrument

In-depth interviews were conducted by three trained research assistants from August to October 2022 with the study participants. A full-day workshop was organized to train the research assistants on data collection protocols. Senior authors supervised the data collection process to ensure consistency and rigor. The interviews which lasted an average of 40 minutes, were conducted using a semi-structured interview guide and audio-recorded to capture participants exact words (47). To ensure validity, the interview guide was pre-tested on four women outside the study area and revisions were made based on the feedback regarding content, structure and question phrasing. Ensuring that the guide was valid, feasible and reliable, led to collecting quality data to address the research questions (48). The interviews took place at participant-selected locations to minimize distractions and allow them to discuss sensitive issues (49). Relevant probes and follow-up questions were utilized to elicit rich, detailed information from participants. Data collection continued until no new information emerged, thus, considering data saturation (50). In this study, code saturation was achieved by the 39^th^ interview as no new information emerged. We conducted 6 additional interviews to confirm saturation. Interviews were conducted in the local dialects and were translated and transcribed each day. Field notes supplemented the transcribed interviews to provide a detailed understanding of the data.

### 2.4 Ethical consideration

Ethics approval was granted by the Fiji National University Human Health Research Ethics Committee (approval number 0009.22). Written informed consent was obtained from all study participants prior to the in-depth interviews. The researchers explained the information sheet and addressed all participants’ concerns. Participants were informed that their involvement was voluntary and could withdraw from the study or decline to answer any interview questions without facing any negative consequences (51). To maintain confidentiality, the researchers assured participants of data anonymity and assigned them unique code to avoid identification (52).

### 2.5 Data management and analysis

The audio recordings and field notes were transcribed by the research assistants, and the investigators verified the accuracy and quality of the transcripts against the original recordings. The data were analyzed thematically in six phases (53). This process of thematic analysis employs a systematic approach to managing qualitative data to enhance greater transparency, credibility, rigor, and validity in the findings (54).

In the first phase of data analysis, two of the researchers thoroughly explored the transcripts through multiple readings to familiarize themselves with the data and search for meanings and patterns (55). The second phase involved generating initial codes where the analysts organized the data into meaningful groups. Thus, the two analysts independently read the transcripts multiple times to identify, organize and code the essential text. The third phase began when all data had been coded and organized. All potentially relevant coded extracts were sorted and collated into themes that reflected participants opinions (56, 57). Some of the codes formed main themes while others led to the formation of sub-themes. In the fourth phase, the investigators reviewed the coded data extracts for each theme to determine whether they formed a coherent pattern.

During the fifth analysis phase, the investigators determined the aspects of the data that each theme captured and identified interesting components and the relevance of each theme. They labeled each theme and determined the story that each communicated. The researchers further determined how each theme fits into the overall story about the whole dataset in relation to the research questions (53). For consistency, themes were considered both independently and in relation to other themes. The final phase of the analysis involved producing the final report, where the researchers communicated the story conveyed by the data in a logical, concise and coherent manner across the established themes and sub-themes. Data extracts in the form of direct quotes from the respondents were used to illustrate the overall story.

Data analysis was inductive, with categories emerging directly from the data (58). The main themes that emerged were the perceived impact of the COVID-19 pandemic on T2DM management, coping strategies and recommended interventions.

### 2.6 Reflexivity, rigor and trustworthiness

The four criteria used to establish methodological rigor and trustworthiness included credibility, confirmability, dependability and transferability (59). The flexibility of the semi-structured interview process enhanced the credibility of the study. Thus, beginning with a broad set of questions and using probes enabled the researchers to obtain in-depth information (60). Audio recordings ensured the accurate capture of participants words during the in-depth interviews. To further enhance the study’s credibility, the researcher established authority in the field, ensured familiarity with the research context, and used experienced qualitative interviewers (61, 62).

The data collection, analysis, and interpretation processes were documented to establish the confirmability criterion. The researchers maintained regular communication to ensure the interviewers strictly followed the study protocol for participant recruitment. The research team systematically reviewed the transcripts against the audio files to check for accuracy, consistency, and clarification (62). The team reached a consensus on the coding process and identified the essential concepts, themes and sub-themes.

Monitoring the data analysis process improved both consistency and results interpretation. To establish dependability, a detailed description of the research methodology was provided, including data collection methods, analysis procedures, and the interpretation of findings (63). The use of purposive sampling ensured a diverse group of participants, thereby satisfying the transferability criterion and facilitating analysis across multiple levels (64).

The study employed reflexivity to ensure quality and rigor, enabling the researchers to examine their roles in knowledge creation and recognize that meanings are situated within specific contexts. It also supported the researchers in assessing and comprehending the ways in which wider socio-cultural contexts impact the research journey (65). We maintained reflexivity by taking reflexive notes during the in-depth interviews, to enrich the data. Rigor, trustworthiness, and ethical integrity were ensured throughout the study.

## 3 Results

A total of 45 in-depth interviews were conducted among T2DM women. Most of the participants were unemployed (40%), married (23%) and belonged to the I-Taukei ethnic group (49%). The majority of them were Christians (42%), from Nasinu (31%) and had lived with T2DM for 6-11years. The age range was 28–62 years (**Table 1**).

**Table 1:** Demographic characteristics of women interviewed.

Study participants (Women)	N=45
Median age in years	**45** (28–62)
Ethnicity	
I-Taukei	**22 (49%)**
Indo-Fijian	18 (40%)
Melanesian	5 (11%)
Marital status	
Married	**23 (51%)**
Single	14 (31%)
Divorced	8 (18%)
Religion	
Christian	**19 (42%)**
Moslem	8 (18%)
Hinduism	14 (31%)
Atheist	4 (9%)
Occupation	
Civil servant	**10 (22%)**
Self-employed	17(38%)
Unemployed	18 (40%)
Number of years living with diabetes	
1-5years	9 (20%)
6-11years	**23 (51%)**
12-17years	13 (29%)
Study settings	
Tamavua	8 (18%)
Nausori	11 (24%)
Lami	12 (27%)
Nasinu	**14 (31%)**

The data analysis revealed three main themes and corresponding sub-themes. The main themes that emerged included the perceived impact of COVID-19 pandemic on T2DM management, coping strategies and recommended interventions (**Table 2**).

**Table 2:** Themes and sub-themes.

Themes	Sub-themes
Perceived impact of COVID-19 pandemic on diabetes management.	Limited access to healthcare workers and diabetes supplies. Canceled or postponed appointments. Change in physical activity. Difficulty in maintaining standard diets. Diabetes complications.
Coping strategies	Emotional eating Religious practices Family support Herbal medications
Recommended interventions	Financial support schemes. Special healthcare for diabetes patients in emergency situations. Intensifying diabetes self-care education Door-to-door diabetes supplies Virtual clinics

### 3.1 Perceived impact of COVID-19 pandemic on diabetes management

#### 3.1.1 Limited access to healthcare workers and diabetes supplies

During the COVID-19 pandemic, many participants struggled to access healthcare as healthcare workers were occupied with managing COVID-related cases. As a result, diabetic patients received less attention and care.

“We those with health issues like diabetes nearly died because health workers were busy taking care of COVID patients which was not good at all. I could not replenish my medications on time which led to increase in my glucose level”. D41

In addition to the difficulties participants faced in accessing medications for glucose level control, many of them expressed dissatisfaction with the way emergency situations were handled within healthcare facilities.

“I felt dizzy during the lockdown, so I called a nurse in the main hospital who told me to come for treatment, but when I got there, they were all busy and I came back home. My situation got worse the next morning, so my sister rushed me to the hospital, and I was given an injection to normalize my glucose. The health workers didn’t treat us well during the pandemic, but we cannot blame them because COVID-19 scared everybody”. D6

“My case was emergency but the nurse I met in the hospital did not give me the care I needed … I was not happy because people with chronic diseases needs more attention since we can easily die from COVID-19”. D35

#### 3.1.2 Cancelled or postponed appointments

Some participants mentioned that their scheduled medical appointments were sometimes postponed or canceled since the onset of the COVID-19 pandemic. This resulted in a lack of confidence in healthcare service delivery and, in some cases, death.

“Do you know that even my appointments to check my glucose level and other things were canceled because of COVID-19? At a point I was confused and didn’t know what to do because I was seriously sick and needed to see the doctor”. D8

“…my sister who was diabetic died because her initial appointment to see her doctor was postponed, which should not be so”. D16

#### 3.1.3 Change in physical activity

The COVID-19 pandemic hindered participants from engaging in their usual physical activities due to fear of contracting the virus. Those who had previously exercised outdoors were unable to continue this practice during the pandemic, which negatively impacted their health outcomes.

“COVID-19 did not help me at all because before the pandemic, I was able to go for a walk every evening, which helped to maintain my sugar level. I could not do this exercise during the pandemic because I feared COVID, and my sugar level increased”. D28

#### 3.1.4 Difficulty in maintaining standard diets

Most of the women identified their inability to maintain their recommended diets as a negative factor hindering diabetes management. This was attributed to a lack of money to purchase nutritious meals, as many had lost their jobs due to the COVID-19 pandemic.

“As a diabetic patient, I know I must eat nutritious foods like fruits and vegetables. My doctor advised me not to take too many sweets, but I lost my job due to COVID-19 and could not follow my diet plan. I have to eat any food available” D40

#### 3.1.5 Diabetic complications

Some participants experienced health problems during the COVID-19 pandemic which complicated their existing health conditions. Many suffered from depression and anxiety due to constantly mourning relatives who died from COVID-19, making it difficult for them to manage their health properly.

“… I don’t think I can forget this experience … My foot was amputated during the pandemic. I’m always crying because COVID killed my husband. I’m now on depression medication and scared of future uncertainties”. D9

“I couldn’t go out to get my diabetes medications to control my glucose level because I was afraid of contracting COVID, which worsened the sore on my finger and led to its amputation.” D25

Participants attributed the complications they experienced during the pandemic to limited information on COVID-19 risk. While some were aware and considered diabetes as an underlying health condition that could place them at increased risk of COVID-19 severity, a few did not believe this.

“I had COVID and nearly died because I’m diabetic. We were told that, diabetes can worsen your condition when you have COVID, and this is what I went through. It took God’s intervention for me to be alive”. (D 11)

“…As for me, I don’t believe what the doctors said about diabetes and COVID-19. My doctor told me to be careful because if I get COVID-19, it can be severe which is not true because I take some herbs to protect me” D43

The study participants identified the perceived impact of COVID-19 on diabetes management which include limited access to healthcare workers and diabetes supplies as well as limited physical activities. Some of the respondents had their health appointments canceled or postponed, and others were unable to continue eating nutritious foods, subsequently contributing to diabetic complications.

### 3.2 Coping strategies

#### 3.2.1 Emotional eating

Most of the women used eating to suppress the negative feelings related to COVID-19, such as loneliness, sadness, stress and fear which disrupted their weight reduction efforts and led to excessive weight gain. Therefore, overeating combined with a lack of physical activity contributed to the uncontrolled weight gain experienced by the study participants.

“COVID-19 is not a good thing because it brought too much pain, sadness, and fear to me. I ate a lot during the lockdown to distract myself from COVID because I was alone. I have now become obese and since I have diabetes, if I don’t monitor my weight, I can experience complications”. D13

Another participant attributed her high blood pressure to uncontrolled weight gain during the COVID-19 lockdown.

“…Before COVID-19, I didn’t have high blood pressure, but now I take blood pressure medications because of COVID … I was eating too many sweets during the lockdown because whenever I feel depressed, I eat more food and have become obese”. D1

#### 3.2.2 Religious practices

Most of the participants gained strength and resilience during the pandemic through religious practices such as prayer, meditation and listening to gospel music. These spiritual activities helped reduce anxiety, fear and sadness caused by COVID-19 and increased their hope for the future.

“…I pray every day and read my Bible to build my hopes and faith in God because He is the only person who can prevent me from getting COVID-19”. D44

“Music and prayers have been my source of strength because everyone is afraid of COVID-19… When I listen to gospel songs, my spirit is lifted, and fears reduces giving me strength in life”. D32

Some of the participants joined online church services for social support and as a means of building resilience to cope during the pandemic.

“We were unable to attend face-to-face church service due to COVID-19, so I occasionally joined online church service. This gave me hope and kept me going during the pandemic”. (D41)

#### 3.2.3 Family support

The bond that resulted from loving relationships with family members, the pleasures of continuing kindness and generosity served as a source of resilience and enabled respondents to cope with the COVID-19 crisis as well as manage their health conditions.

“I thank my family members for their support because it is what is keeping me going since I lost my job due to COVID-19. My sisters and brothers send me food and money to sustain me and that really helped me a lot”. D1

#### 3.2.4 Herbal medications

During the COVID-19 pandemic, some women used herbal medicines to manage their glucose levels. While some preferred herbal medications, others opted for them because health care workers were not always available to attend to them.

“I was drinking boiled herbs every day because it’s very good for diabetes. My grandmother showed me that herb before she died. I like our traditional medicines more than those given in the hospital”. D17

Participants who used herbal medicines due to limited support from health care providers explained their situations during the in-depth interview as follows.

“As for me I had no option but to use herbal medicine to manage my sugar levels … the nurses were busy taking care of COVID patients. They didn’t attend to me, so I decided not to go there again”. D23

The coping strategies used included emotional eating, where some of the women used food as a coping mechanism. Religious practices also provided most of the participants with comfort, hope and resilience for a better tomorrow. While some relied on their families for financial support, others relied on herbal medication, which worsened their health conditions.

### 3.3 Recommended interventions

#### 3.3.1 Financial support schemes

Participants recommended that the government institute financial schemes to support diabetes patients financially during emergency situations, such as the COVID-19 pandemic. This could enable them to purchase nutritious foods to improve their health, especially during economic crises.

“I think the government should provide us with money in situations like this because some of us have lost our jobs making it difficult to eat well…”. D34

“To be frank with you, it’s difficult for me because I lost my job due to COVID-19. I cannot follow my doctor’s advice to eat more vegetables and fruits to maintain my sugar level because I don’t have money. It would be good for the government to provide us with some money for food.” D19

#### 3.3.2 Special healthcare services for diabetes patients in emergency situations

During the COVID-19 pandemic, healthcare professionals were busy. As such, participants suggested that special healthcare provisions should be made for diabetic patients during emergency situations to avoid complications and death. This includes allocating healthcare professionals to take care of them and respond to their calls when they cannot be physically present.

“I think the Ministry of Health should assign specific health workers to care for individuals with diabetes during situations like the COVID-19 pandemic to prevent health complications since my mother was diabetic died during the pandemic”. D3

#### 3.3.3 Intensifying diabetes self-care education

The women in the study area have identified the need for health workers to intensify diabetes self-care education. According to participants, receiving the right information on diabetes management would empower them to manage the disease during emergency situations.

“I think they should teach us everything about diabetes management because I nearly killed myself by taking too many drugs. If they teach us and there is a pandemic, we won’t depend on them too much.” D45

“I used herbal medicines because I didn’t know what to do during the COVID-19 lockdown. My aunt told me that herbal medicines can prevent me from getting COVID and lower my glucose level since it had increased”. D17

Most of the women raised concerns about the lack of diabetes-specific COVID-19 information from healthcare workers as such information could be trusted compared to what the general population provided. During the pandemic, many of the participants preferred information from healthcare workers over what the public including the media provided. Some received misleading information from the media causing anxiety and fear. Participants explained the situation as follows.

“As you can see, there is too much misleading information in the media about how COVID-19 affects diabetes patients and how we can stay safe, causing fear and panic. I wish healthcare workers can educate us on how to manage diabetes to prevent complications and death because I trust them”. D8

“In a situation like this, it would be good to prevent the media from providing misleading information…”. D18

#### 3.3.4 Door to door diabetes supplies

The study participants recommended a door-to-door supply of diabetes medications and other necessary supplies to help maintain blood sugar levels, prevent serious complications and reduce deaths.

“The best thing the government can do for us is to provide us with glucometers, strips and medications in our homes during the pandemic to improve our health. My sister died because her diabetes medications ran out, and we could not replenish them”. D6

#### 3.3.5 Virtual clinics

Participants suggested the institution of virtual clinics to assist diabetic patients during emergency situations, due to limited access to healthcare workers during the COVID-19 pandemic.

“I think the health workers should organize online consultations for us because diabetes is killing many women here. The health workers can talk to us on phone and show us how to manage our glucose level. I went to the hospital, but no health worker attended to me, and this nearly caused my death”. D25

Though most women preferred face-to-face appointments, many explained that virtual clinics such as telephone or online communication during the COVID-19 pandemic could have helped to address the issue of delayed access to healthcare. This could have improved patients’ health status and increased their digital literacy.

“Face-to-face consultation is good and I prefer that, but we could have also talked to the health workers on the phone or using online means like Zoom during the pandemic. This is better than nothing because health workers can still listen to your condition and prescribe medication. In that way, we can learn how to use Zoom and other online systems to seek care. It will also prevent us from contacting COVID-19”. D11

The recommended strategies identified by the study participants included the provision of financial support and health care providers specifically for diabetes patients. The need to intensify diabetes self-care education, virtual clinics, home supplies of diabetes medications and other essential supplies could minimize complications and improve the health of diabetes patients.

## 4 Discussion

### 4.1 Perceived impact of COVID-19 pandemic on T2DM management

The study’s findings reveal limited access to healthcare workers and diabetes supplies during the COVID-19 pandemic, which could have contributed to worsening the health conditions of women in the study area (66, 67). Timely interventions including lifestyle changes, the use of effective and affordable medicines and diagnostic tests have been established to reduce morbidity and mortality related to diabetes. However, these essential interventions are not always available in low- and middle-income countries like Fiji (30, 68). The COVID-19 pandemic compounded the situation by interrupting access to regular medical care, with most healthcare professionals assigned to COVID-19 related cases and in person appointments considerably reduced (69–71).

The cancellation or postponement of diabetes appointments during the pandemic contributed to increased diabetic complications among women in the study area, which is consistent with other studies (72, 73). This led to delays in diagnosis, treatment, and management of diabetes in women, putting them at a higher risk of complications (74, 75). Therefore, it is important to implement policies that ensure timely access to healthcare services for diabetic women, especially during emergencies. As the COVID-19 pandemic spread around the world in 2020, most countries recorded a decrease in healthcare services for non-COVID-19-related diseases like diabetes mellitus (76). Specific routine health check-ups among T2DM patients revealed a decrease in the proportion of patients who obtained HbA1c testing (77). Cancellation of in-person appointments and patients fear of becoming infected while attending clinics worsened their health conditions (78). This led to complications including diabetic foot amputations among the study participants. This situation is consistent with a survey, that found that HbA1c values significantly worsened among patients as a result of the reduction in HbA1c testing during the COVID-19 pandemic (79). Research has identified more severe and acute presentations of diabetes-related foot disease, along with higher amputation rates, during the pandemic compared to the pre-pandemic period (80).

Lifestyle changes resulting from the COVID-19 pandemic such as a decrease in physical activity and the inability to maintain a standard diet, negatively impacted the management of T2DM among women in the study area. Participants inability to engage in physical activities to control their glucose levels during the pandemic is consistent with other studies (21, 81). Diabetic complications experienced by the study participants during the pandemic could partly be attributed to a reduction in the level of physical activity and an increased rate of sedentary behavior during the COVID-19 pandemic (82).

Financial constraints from pandemic-related job losses hindered most of the participants from purchasing nutritious food. These employment disruptions led to changes in dietary habits, forcing compromised nutritional quality (83, 84). Other studies have linked unhealthy dietary patterns during the pandemic to limited financial resources to purchase quality foods (85–87). Given that a significant proportion of deaths in Fiji are attributed to diet and lifestyle factors, the inability to access nutritious food during the pandemic poses a risk of increasing T2DM related mortality (88–90). Improving the dietary patterns among women with diabetes could enhance their overall health and extend their life expectancy (91, 92).

### 4.2 Coping strategies

Emotional eating, which served as a coping mechanism for women in the study area to manage the psychological distress associated with the pandemic, led to weight gain and worsened health. The impact of COVID-19 preventive measures such as working from home and avoiding social gatherings, on weight is crucial for individuals with diabetes, as it can change their dietary behaviors (93–95). Diabetic women who eat too much to cope with COVID-19 related stress and anxiety, could worsen their health status. Therefore, it is important for these women to prioritize weight management to improve their health (96, 97). This study expands our understanding of the influence of the COVID-19 pandemic on the dietary behaviors and nutritional challenges faced by women living with T2DM.

The culture of communal living and maintaining healthy relationships with friends and family, built resilience among women with T2DM during the pandemic. Family members exhibited a high sense of responsibility by assisting with food and money for their daily upkeep. Being responsible for one another is a good thing since it increases a sense of belonging, which could improve the health and well-being of individuals (98). Various studies have highlighted the important role that diabetic self-practices play in enhancing outcomes, such as maintaining physical activities, eating healthy foods, monitoring blood glucose levels, complying with a medication regimen, improving psychosocial health and embracing risk-reduction behaviors such as avoiding drinking and smoking (99–102). Nonetheless, the support offered by family members could be linked to greater adherence to diabetes self-management (103), as identified by this study findings.

Religious, cultural, and spiritual beliefs influence diabetes management in Fiji (103, 104). While these beliefs can occasionally delay clinical intervention by fostering an overdependence on religious or traditional healers (105, 106), they also shape how individuals perceive and manage chronic diseases like T2DM (107). In Fiji’s conservative society, these cultural frameworks play a critical role in determining how women with diabetes are perceived and supported by their families and the wider community. Ultimately, integrating spirituality into diabetes management can improve outcomes by leveraging these belief systems to build resilience and strengthen social networks (108, 109). Research has identified that individuals who partake in religious practices tend to have lower HbA1c levels and obtain emotional support as a coping strategy (110). Likewise, other scholars have revealed that greater social support and social involvement in diabetes care are associated with better diabetes self-care management (111, 112). Though in-person religious activities were not possible during the pandemic, participants involvement in online church activities, personal devotions and prayers enabled them to build resilience.

The study findings on the use of herbal medicines align with insights from prior literature indicating that traditional remedies are often integrated into religious practices for managing T2DM (113, 114), with many patients considering them as God-given remedies (113). While these herbs provided a necessary coping mechanism during the pandemic, others maintained a balanced trust in both traditional and conventional medicine, believing doctors are also divinely inspired (115). Although herbal medications served as a coping strategy for participants, their potentially adverse effects highlight the need for further research to assess the safety and efficacy of traditional remedies used in Fiji (116).

### 4.3 Recommended interventions

Participants’ recommendations for financial support are essential for improving the health of women who lost their income during the pandemic and could not afford prescribed diets. Addressing financial barrier is crucial, as optimized nutrition can mitigate insulin resistance and pancreatic beta-cell dysfunction, ultimately enhancing glycemic control (117). Financial constraints often prevent patients from purchasing medications, a problem intensified in areas relying on out-of-pocket payments. While free medication schemes exist, logistical barriers like mandatory in-person pickup can still hinder access, underscoring the need for the home delivery models suggested by the study participants.

Furthermore, nutrition-focused interventions that address eating patterns, meal timing, nutrient composition, and food choices can improve overall and postprandial glycemic control, effectively maintaining blood glucose levels within therapeutically established target ranges (118, 119). Diet changes are pivotal to managing T2DM, essential for achieving long-term glycemic stability (119). In low socioeconomic areas, food security and homelessness due to the inability to pay rent are among the social factors that can exacerbate the effects of a pandemic in people with pre-existing health conditions such as diabetes (120). The study findings highlight the need to establish financial support schemes for diabetic women in the study area, to maintain healthy dietary patterns and reduce the risk of complications and death.

Providing special health care services for women with diabetes during emergencies is considered essential, as many healthcare workers prioritize COVID-19 cases, leaving routine diabetic care largely unattended. Given the increased cases of T2DM and obesity among women in Fiji (27) and diabetic complications (29, 30), neglecting diabetic care during emergencies, as identified in this study, can compound an already critical public health issue. Healthcare professionals managing diabetic patients across Europe and the United Kingdom, reported major service disruptions (121). Another study in the United States of America reported substantial deficiencies in routine diabetes care (122). Similarly, a survey of healthcare professionals across 47 nations, including low and middle income countries identified diabetes as the chronic condition most affected by the pandemic due to widespread care disruptions (123).

Participants suggestions for virtual clinics could improve diabetes care during emergencies. In many high-income countries, the pandemic prompted a shift from in-person visits towards technological support for the self-management of chronic diseases including diabetes. A global survey across 27 countries showed a significant increase in virtual consultations with individuals living with diabetes mellitus. Healthcare providers used telephone, email, and video platforms to maintain contact with patients (124). While most of the women in this study preferred face-to-face consultations, they also recognized the need for virtual consultations during the pandemic. For some, this was an opportunity to improve their digital literacy skills and avoid contacting the COVID-19 virus. Given that a large proportion of Fiji’s population resides in hard-to-reach areas, introducing online consultation platforms could improve the management of diabetes and patients’ health outcomes. Fiji serves as the digital hub for the Pacific, supported by advanced subsea cables and expanding mobile networks. However, challenges in rural internet connectivity and infrastructure persist. This highlights the need for a strong commitment from the government and relevant stakeholders to enhance infrastructure for online consultations (125).

The study findings on intensifying diabetes self-care education can enable participants to maintain optimal blood glucose levels, boost their immune system and reduce the risk of COVID infection and severe outcomes (126). Diabetes self-management education is crucial and has the potential to mitigate negative lifestyle behaviors and emotional well-being during the pandemic (127, 128). Providing the right information to T2DM patients could minimize their overreliance on traditional medicines and correct COVID-19 misconceptions as some were unaware that diabetes could place them at increased risk of COVID-19 severity. Other scholars have revealed the high level of trust the general public has for healthcare workers regarding the accuracy of information on COVID-19 (129). Delays in the delivery of self-management education programs are a major concern, as these programs have been shown to have positive benefits on both physical and psychological health (53).

## 5 Conclusion

The pandemic caused a significant interruption to T2DM diabetes care, characterized by severely limited access to healthcare professionals and essential diabetes supplies. This interruption, driven by canceled or postponed health appointments and staff deployment to COVID-19 wards, led to poorer glycemic control and increased diabetes complications among women with T2DM, in the study area. Establishing systems that provide continuity of care for diabetic women during emergency situations is crucial and requires a multifaceted approach that integrates preparedness, sustainable and coordinated healthcare services. Such care could be obtained through virtual clinics, readily accessible specialized healthcare services and providing diabetes supplies to women at their residences.

Educating women to prioritize weight management through engaging in physical activities and nutritious eating is crucial to reduce complications of type-2 diabetes and improve the health and well-being of women with T2DM in the study area. Financial constraints which hinder the consumption of nutritious foods among participants, necessitate the provision of unemployment benefits to assist women facing financial difficulties in times of crisis. Strengthening family support networks and participating in religious activities are recognized as important protective factors that foster resilience against financial difficulties, loneliness and depression, especially during public health disasters like the COVID-19 pandemic. Healthcare managers, healthcare workers and community leaders must educate women on the potential risks associated with emotional eating, unsupervised use of herbal medicines and type-2 diabetes management to prevent obesity, metabolic disorders and severe health consequences.

The findings of this study have implications for public health and upcoming research in the face of future pandemics. There is a need for systemic, proactive and equity-oriented approaches to establish decentralized digital care models and provide psychosocial support for women with T2DM. The study has provided baseline information for further studies and underscores the need for research to ascertain the feasibility of establishing virtual clinics in Fiji to ensure continuity of care in emergency situations. Additional interventions to provide mental health support to prevent diabetes distress among women in the study area are crucial.

## 6 Study limitation

Though the sample size used was sufficient to reach saturation, the findings cannot be generalized due to the diverse opinions of individuals across different localities. Another limitation is the potential for recall bias, as participants relied on their own memories to describe past experiences. Nevertheless, it has provided detailed contextual information on the phenomenon investigated.

## Data Availability

The original contributions presented in the study are included in the article/supplementary material. Further inquiries can be directed to the corresponding author.
